# A case of repeat resection for recurrent pulmonary metastasis from sebaceous gland carcinoma

**DOI:** 10.1186/s40792-020-00947-1

**Published:** 2020-08-12

**Authors:** Sachi Kawagishi, Ryu Kanzaki, Seiji Taniguchi, Kenji Kimura, Toru Kimura, Hiroyuki Takabatake, Eiichi Morii, Masayoshi Inoue, Yasushi Shintani

**Affiliations:** 1 Department of General Thoracic Surgery, Osaka University Graduate School of Medicine, 2-2 (L5), Yamadaoka, Suita, 565-0871 Japan; 2 Department of General Thoracic Surgery, Osaka International Cancer Institute, Osaka, Japan; 3 Department of Thoracic Surgery, Suita Municipal Hospital, Suita, Japan; 4 Department of Pathology, Osaka University Graduate School of Medicine, Suita, Japan; 5 Division of Thoracic Surgery, Department of Surgery, Kyoto Prefectural University of Medicine, Kyoto, Japan

**Keywords:** Sebaceous gland carcinoma, Pulmonary metastasis, Surgery

## Abstract

**Background:**

Sebaceous gland carcinoma (SGC) of the eyelid is an aggressive malignant eyelid tumor, and it can metastasize to the regional lymph nodes and distant organs. There have been only a few reported cases of patients who underwent pulmonary metastasectomy for metastatic SGC. We herein report a patient who underwent repeat pulmonary metastasectomies for recurrent pulmonary metastases from SGC.

**Case presentation:**

Bilateral small pulmonary nodules were detected in a 59-year-old woman with a history of eyelid SGC. She underwent wide wedge resection of the left lower lobe, and the disease was diagnosed as pulmonary metastases from SGC. Six months after the first pulmonary resection, CT showed that the nodules of right S2 and S10 had increased in size, and three small nodules had newly appeared in the right lung. The patient therefore underwent six wide wedge resections of the right lung through thoracotomy. After that, she underwent pulmonary metastasectomy 2 times. Ninety months after the first pulmonary resection, the patient is doing well without disease.

**Conclusions:**

Given that a long-term survival was ultimately achieved in the present case, repeat pulmonary metastasectomy may be beneficial for recurrent pulmonary metastasis from SGC.

## Background

Malignant eyelid tumors account for 5–10% of all malignant skin tumors [1]. Sebaceous gland carcinoma (SGC) of the eyelid is an aggressive malignant eyelid tumor and can metastasize to the regional lymph nodes and distant organs. There is currently no standard treatment strategy for recurrent pulmonary metastasis of SGC, because of the rarity of the disease. There have only been a few reports of patients who underwent pulmonary metastasectomy due to metastatic SGC. Furthermore, to our knowledge, there have been no reports on repeat resection for recurrent pulmonary metastasis of SGC. We herein report the case of a patient who underwent repeat pulmonary metastasectomy for recurrent pulmonary metastasis from SGC.

## Case presentation

**Fig. 1:**
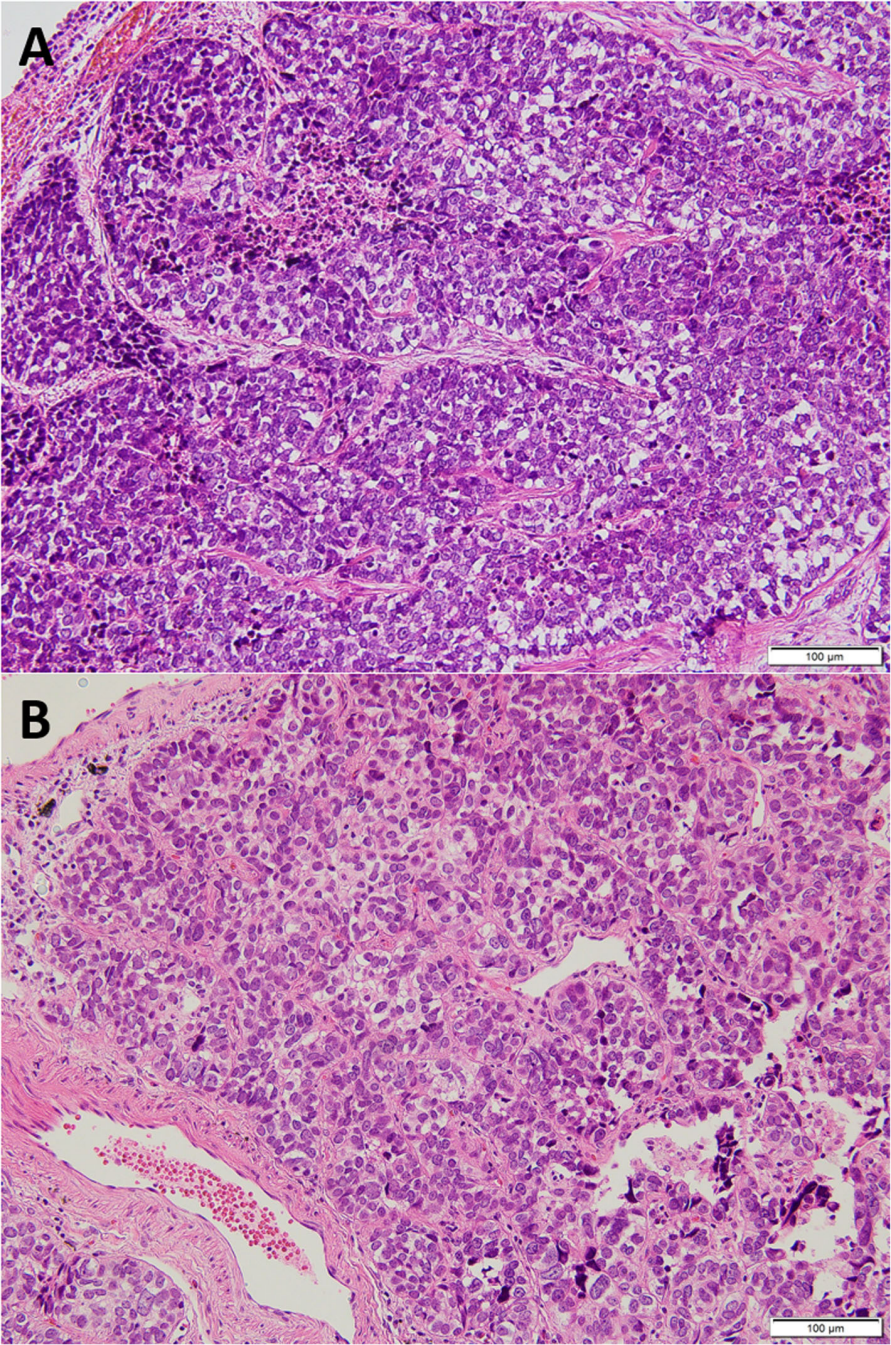
**a** HE staining of the eyelid tumor. Tumor cells with atypical nuclei and foamy cytoplasm expanded to form a net-like structure (magnification × 200). **b** HE staining of the pulmonary lesion. The pathological findings were similar to those of the primary tumor (magnification × 200)

**Fig. 2:**
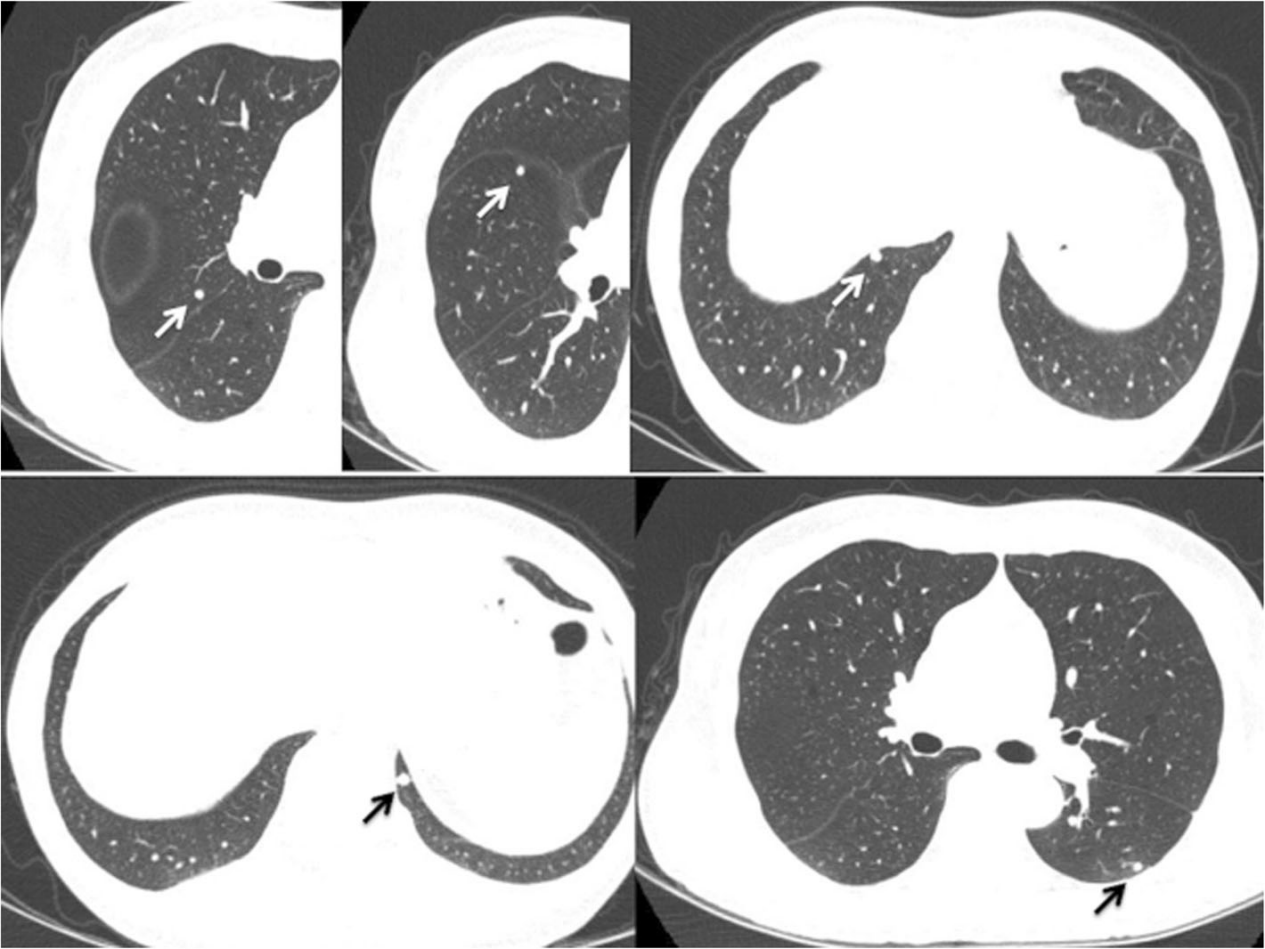
Chest CT findings. Small pulmonary nodules located in S6 and S10 of the left lung and S2, S4, and S10 of the right lung

A 59-year-old woman was referred to our department due to an abnormal shadow on chest computed tomography (CT). She had undergone right lower lid resection for a tumor of the eyelid 4 years earlier. The size of the lesion had been 11 mm, and HE staining showed that the tumor cells with atypical nuclei and foamy cytoplasm had expanded to form a net-like structure. The pathologic diagnosis was SGC (Fig. 1a). Two years after the first operation, she suffered from local relapse of SGC and underwent right lower lid resection for the tumor of the eyelid and radiotherapy with a total dose of 60 Gy of proton beam therapy after the operation. Four years after the first operation, she suffered from a recurrent subcutaneous tumor of the right lower eyelid and underwent orbital exenteration of the right eye. At the same time, chest CT revealed bilateral small pulmonary nodules located in segment (S) 6 and S10 of the left lung and S2, S4, and S10 of the right lung (Fig. 2). A preoperative pulmonary function test revealed normal results (FEV1.0, 2600 ml; %VC, 123.6%).

**Fig. 3:**
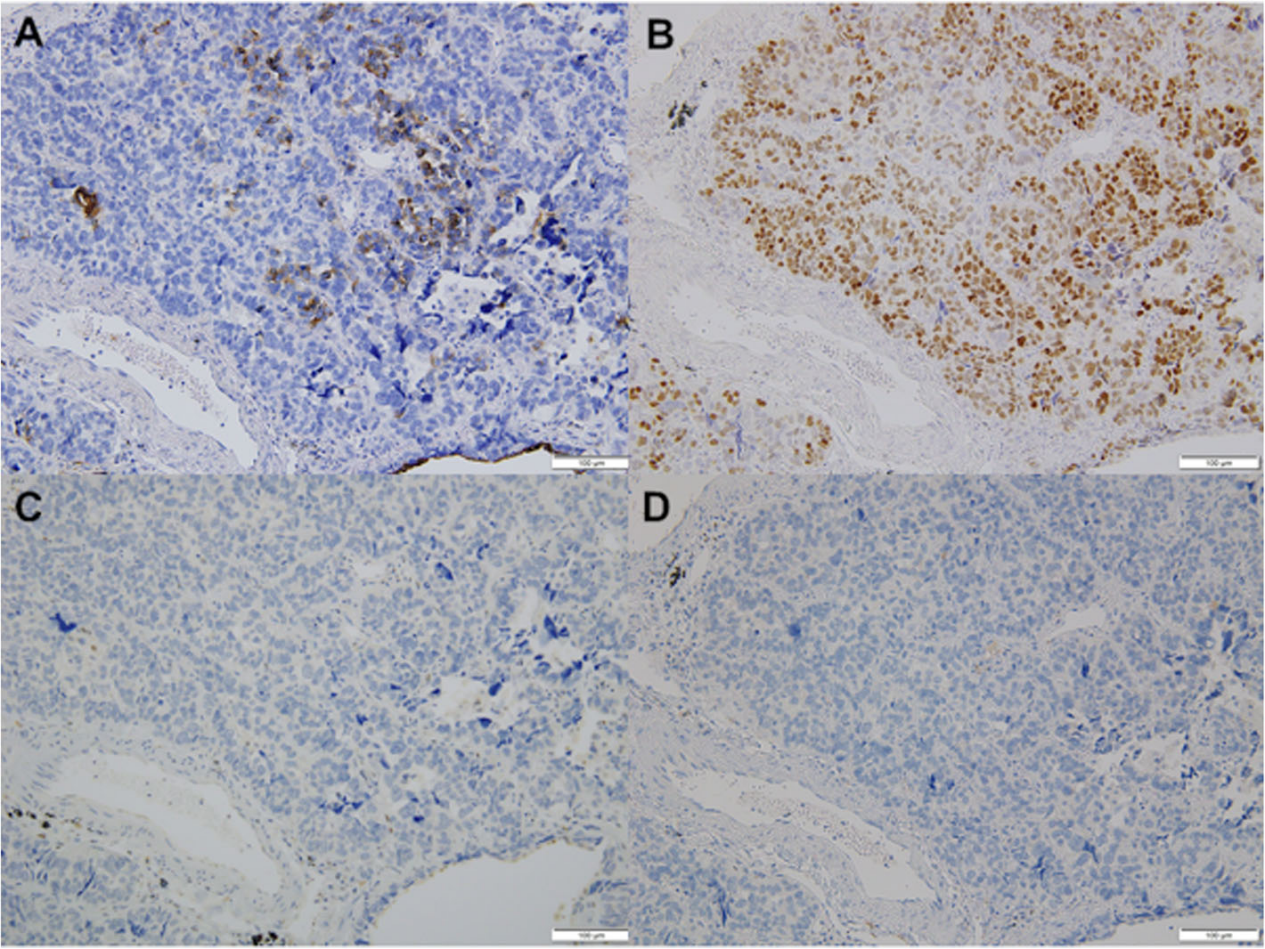
Results of an immunohistochemical analysis of the pulmonary disease. **a** EMA, **b** AR, **c** p53, **d** Ber-EP4 (magnification × 200)

Because the lung nodules were small, transbronchial or CT-guided biopsy was not indicated. We therefore decided to perform surgical biopsy. At the same time, we planned staged radical surgery for all nodules in both lungs in the event that the final diagnosis was pulmonary metastasis. She underwent two wide wedge resections of the left lower lobe (S6, S10). The tumor size of S6 was 8 mm × 4 mm, while that of S10 was 5 mm × 4 mm, and the surgical margin was negative. HE staining showed that pathological findings are similar to primary tumor (Fig. 1b). An immunohistochemical analysis revealed that the tumor expressed epithelial membrane antigen (EMA), androgen receptor (AR), and p53 and not expressed anti-epithelial antigen (BerEP-4) (Fig. 3). The primary tumor also showed the same pattern of immunohistochemistry. The disease was therefore diagnosed as pulmonary metastasis from SGC of the eyelid.

After the operation, 18F-fluorodeoxyglucose positron emission tomography/CT (FDG-PET/CT) was performed, which showed no metastasis other than that at the right lung. The remaining small nodules in the right lung did not show any uptake of FDG. Six months after the first pulmonary resection, CT showed that the nodules of right S2 and S10 had increased in size, and three small nodules had newly appeared at S3, S6, and S8 of the right lung. The patient therefore underwent six wide wedge resections of the right lung (S2, 3, 4, 6, 8, 10) through thoracotomy. The pathologic diagnosis was pulmonary metastasis from SGC of the eyelid.

Eighteen months after the first pulmonary resection, CT revealed a new nodule 7 mm in diameter in the left S1+2. She underwent wide wedge resection of the left upper lobe through thoracotomy. Forty-nine months after the first pulmonary resection, she underwent wide wedge resection of right lung to again treat recurrent pulmonary metastasis. The postoperative pulmonary function remained almost unchanged from the preoperative pulmonary function (FEV1.0, 2520 ml; %VC, 110.3%). Ninety months after the first pulmonary resection, the patient is doing well without disease.

## Conclusions

SGC is a rare and potentially aggressive malignancy. Most SGC tumors are located in the extraocular head and neck skin (face/ear/scalp/neck/lip, 42.8%), followed by the eyelid (34.5%), trunk (14.8%), and extremities (6.5%). Ocular SGC arises from the sebaceous glands, whereas the origin of extraocular sebaceous carcinoma has not been determined [2]. SGC of the eyelid is a malignant eyelid tumor that is derived from sebaceous glands, such as the meibomian gland and Zeis gland. The proportion of histologic types of eyelid tumors differs among ethnicities. In the USA and Europe, basal cell carcinoma (BCC) accounts for 80% of all eyelid tumors, followed by squamous cell carcinoma (SCC), and SGC accounts for < 5% of all eyelid tumors [3–5]. In contrast, it is reported that SGC of the eyelid is as prevalent as or even more common than BCC in Asian countries, including Japan [3, 6]. The clinical appearance of ocular SGC is highly heterogeneous and often mimics other ocular benign conditions, such as chalazion, posterior blepharitis, superior limbic keratoconjunctivitis, and keratitis [5]. Thus, the diagnosis might be delayed, resulting in a poor outcome.

The overall mortality rate of eyelid SGC is 5–10%. It is reported that metastatic sites of SGC include the regional lymph nodes, liver, brain, bone, and lung. Regional lymph node metastasis was noted in 20% of all cases and systemic metastasis in 14% [6]. The duration from the diagnosis of primary SGC to metastasis may range from 0 to 62 months [7]. In the present case, pulmonary metastasis was discovered 4 years after the operation of primary SGC. Based on our experience, we propose that long-term follow-up for at least 5 years be performed after surgery for primary SGC.

The diagnosis of SGC of the eyelid is made based on the pathologic diagnosis of specimens from surgical resection or a biopsy. Pathologic findings of SGC vary widely, ranging from well-differentiated tumors to undifferentiated tumors. The diagnosis of SGC is generally based on HE staining and Oil Red O staining for lipids [4, 5]. Recently, it was reported that an immunohistochemical analysis can replace Oil Red O staining, helping to differentiate primary SGC from BCC and SCC [5]. In the present case, both the primary SGC and pulmonary metastases were positive for EMA, AR, and p53 and negative for BerEP-4 according to an immunohistochemical analysis. Based on these findings, the pulmonary disease was diagnosed as pulmonary metastasis from SGC.

The standard treatment strategy for recurrent pulmonary metastasis of SGC has not been established. Evidence to support systemic therapy for the treatment of sebaceous carcinoma with curative or palliative intent is confined to case reports. The majority of regimens include 5-fuorouracil or cisplatin-based chemotherapy [8]. Recently, the administration of cetuximab or pembrolizumab for the treatment of metastatic SGC has been reported [9, 10]. However, level of evidence in these reports was not high; thus, it is said that there are no promising systemic treatments. Because pulmonary metastasectomy is an established treatment for resectable pulmonary metastasis [11], pulmonary metastasectomy was considered as the most appropriate mode of treatment in the present case. On the other hand, stereotactic body radiotherapy (SBRT) for pulmonary metastasis could be an alternative to pulmonary resection. Although it is reported that SBRT provides a favorable 3-year local control rate for pulmonary metastasis from epithelial tumors [12], there have been no reports on the use of SBRT in the treatment of pulmonary metastasis from SGC. Based on these findings and the fact that the treatment was considered tolerable based on the patient’s good pulmonary function, we performed repeat pulmonary resection.

Thus far, only two cases in which surgical resection was performed for pulmonary metastasis from SGC have been reported [7, 13]. One case was pulmonary metastasis of the right upper lobe that was found 6 years after the operation of left primary SGC. The patient underwent wide wedge resection of the right upper lobe, and 7 months after pulmonary operation, she is doing well without disease. Another case was pulmonary metastasis of the left upper and lower lobes found 5 years after the operation of right primary SGC. Following neoadjuvant chemotherapy, the patient underwent wide wedge resection of the left upper and lower lobes. Three months after pulmonary resection, she is doing well without disease. Both cases are similar to our own in that pulmonary metastasis was detected during long-term follow-up after an operation for primary SGC.

Repeat pulmonary metastasectomy is reportedly beneficial in many types of cancer [11]. Our case underwent four pulmonary metastasectomies for recurrent pulmonary metastasis from SGC. To our knowledge, the present case is the first to undergo repeat pulmonary metastasectomy for recurrent pulmonary metastasis of SGC. Given that a long-term survival was ultimately achieved in the present case, repeat pulmonary metastasectomy may be beneficial for recurrent pulmonary metastasis from SGC.

## Data Availability

Data sharing is not applicable to this article as no datasets were generated or analyzed during the current study.

## Abbreviations

CT: Computed tomography
FDG: 18F-fluorodeoxyglucose
SGC: Sebaceous gland carcinoma
FEV1.0: Forced expiratory volume in 1 s
%VC: Vital capacity percentage
